# Supplementary material for: Blockchain and explainable-AI integrated system for Polycystic Ovary Syndrome (PCOS) detection
Source: PeerJ Comput Sci. 2025 Feb 28;11:e2702. doi: 10.7717/peerj-cs.2702 (PMC11888934; doi:10.7717/peerj-cs.2702)
Supplement: Supplemental Information 3 [file peerj-cs-11-2702-s003.docx]

=================================================================

Follow the below-mentioned instructions to set up the running Environment:

1. Install the prerequisites to run hyperledger fabric blockchain in Ubuntu OS- Go, Git, cURL, Docker.

2. Clone the hyperledger/fabric-samples repository available at https://github.com/hyperledger/fabric-samples.

3. install-fabric.sh script in the fabric-samples automates the process to set up your environment. Use comments below.

$curl -sSLO https://raw.githubusercontent.com/hyperledger/fabric/main/scripts/install-fabric.sh && chmod +x install-fabric.sh

$./install-fabric.sh d s b

4. Under fabric-samples/chaincode/lib, upload the fabric_code.js file

5. Install Jupyter notebook

6. Load code.ipynb in the Jupyter Notebook and upload the PCOS dataset from the Kaggle website.

===================================================

Run the hyperledger fabric network

$cd fabric-samples/test-network

$./network.sh up

$./network.sh createChannel

$./network.sh deployCC -ccn hie -ccp ..../chaincode -ccl javascript

$export PATH=${PWD}/../config/.

$export FABRIC_CFG_PATH=$PWD/.../config.

# Environment variables for Org1

$export CORE_PEER_TLS_ENABLED=true

$export CORE_PEER_TLS_=true

$export CORE_PEER_TLS_ROOTCERT_FILE=${PWD}/organizations/peerOrganizations/[org1.example.com/peers/peer0.org1.example.com/tls/ca.crt](http://org1.example.com/peers/peer0.org1.example.com/tls/ca.crt)

$export CORE_PEER_MSPCONFIGPATH=${PWD}/organizations/peerOrganizations/[org1.example.com/users/Admin@org1.example.com/msp](http://org1.example.com/users/Admin@org1.example.com/msp)

$export CORE_PEER_ADDRESS=localhost:7051

peer chaincode invoke -o localhost:7050 --ordererTLSHostnameOverride orderer.example.com --tls --cafile

"${PWD}/organizations/ordererOrganizations/example.com/orderers/orderer.example.com/msp/tlscacerts/tlsca.example.com-cert.pem" -C mychannel -n mlcode -peerAddresses localhost:7051 --tlsRootCertFiles "${PWD}/organizations/peerOrganizations/org1.example.com/peers/peer0.org1.example.com/tls/ca.crt" --peerAddresses localhost:9051 --tlsRootCertFiles "${PWD}/organizations/peerOrganizations/org2.example.com/peers/peer0.org2.example.com/tls/ca.crt" -c '{"function":"initLedger","Args":[]}'

Run the code.ipynb Notebook in the Jupyter notebook

==================================================================
